# Supplementary material for: Lamin A molecular compression and sliding as mechanisms behind nucleoskeleton elasticity
Source: Nat Commun. 2019 Jul 11;10:3056. doi: 10.1038/s41467-019-11063-6 (PMC6624373; doi:10.1038/s41467-019-11063-6)
Supplement: Supplementary file 1 — Supplementary Information [file 41467_2019_11063_MOESM1_ESM.pdf]

SUPPLEMENTARY INFORMATION

Lamin A molecular compression and sliding as mechanisms behind nucleoskeleton elasticity

Alex A. Makarov, Juan Zou, Douglas R. Houston, Christos Spanos, Alexandra S. Solovyova, Cristina Cardenal-Peralta, Juri Rappsilber, and Eric C. Schirmer

Supplementary Figures

Supplementary Discussions

Supplementary Tables

Supplementary Note 1

Supplementary References

Supplementary Figures

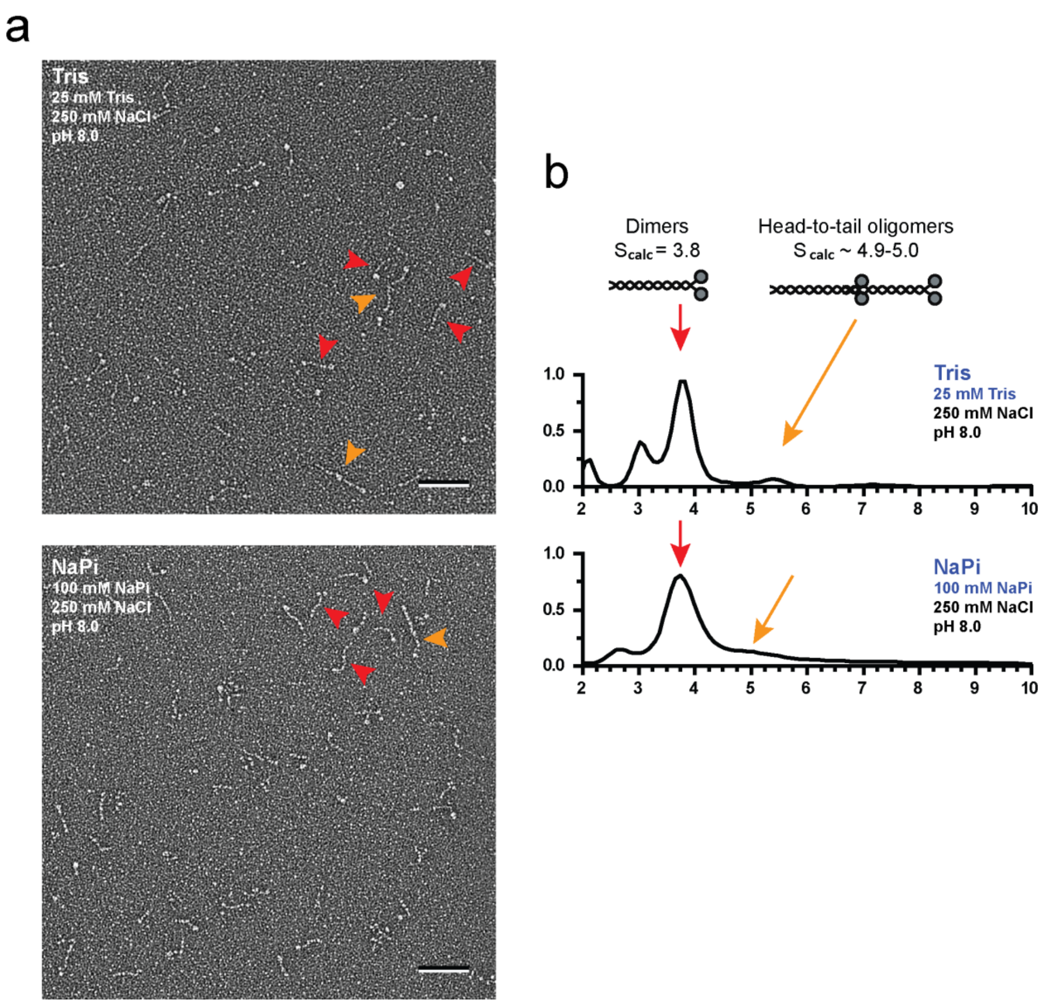

Supplementary Fig. 1. Lamin A oligomeric state in Tris and NaPi buffers. a EM micrographs of rotary metal shadowed lamin A equilibrate in either Tris or NaPi buffer show predominant oligomeric state to dimers (red arrowheads) with a few head-to-tail tetramers (orange arrowheads, ~ 1 tetramer per 20 dimers in NaPi buffer, see Source Data file). Scale bars, 100 nm. **b** Analytical ultracentrifugation captures clearly separable population of dimers and head-to-tail tetramers with S-values closely matching values calculations in SoMo using dimer and mock tetramer models: 3.8 S and 4.9-5.0 S respectively (see Source Data file).

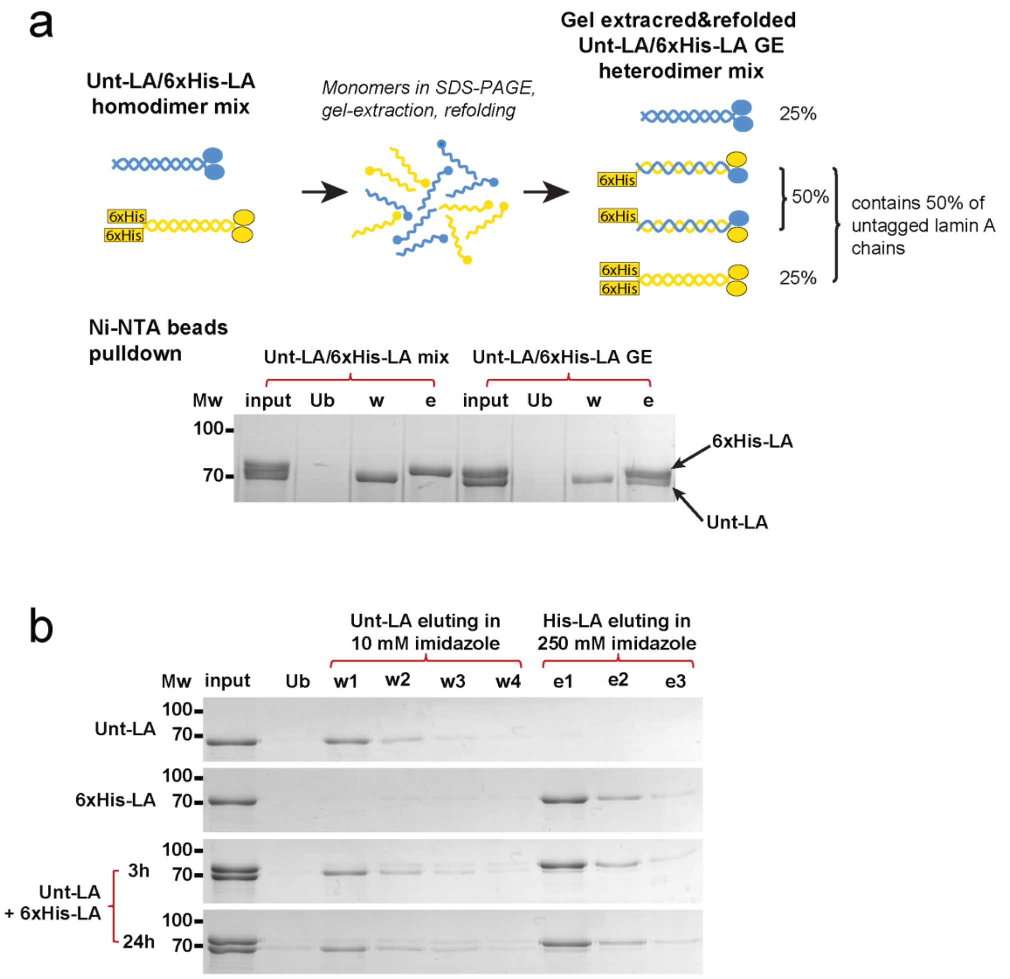

Supplementary Fig. 2. Lamin A dimer chain exchange assay. a Co-gel extraction of untagged and 6xHis-tagged lamin A dimers results in homo/hetero-dimer mix: affinity pull down of untagged (Unt-LA) and 6xHis-tagged (6xHis-LA) lamin A dimers, mixed immediately prior, shows separate elution of the two populations of dimers – in 10 and 250 mM imidazole (**w** and **e** respectively); after gel-extraction ~50% of untagged lamin A elutes with 6xHis-tagged lamin A in 250 mM imidazole. **b** Prolonged co-incubation of untagged and 6xHis-tagged lamin A dimers does not result in sporadic chain exchange: affinity pull down of untagged lamin A, 6xHis-tagged lamin A or a mix of these (Unt-LA + 6xHis-LA), pre-incubated for 3 or 24 h indicates that lamin A dimers do not exchange chains in 6 M urea: untagged and His-tagged lamin A dimers always elute separately from the nickel column (two bottom rows).

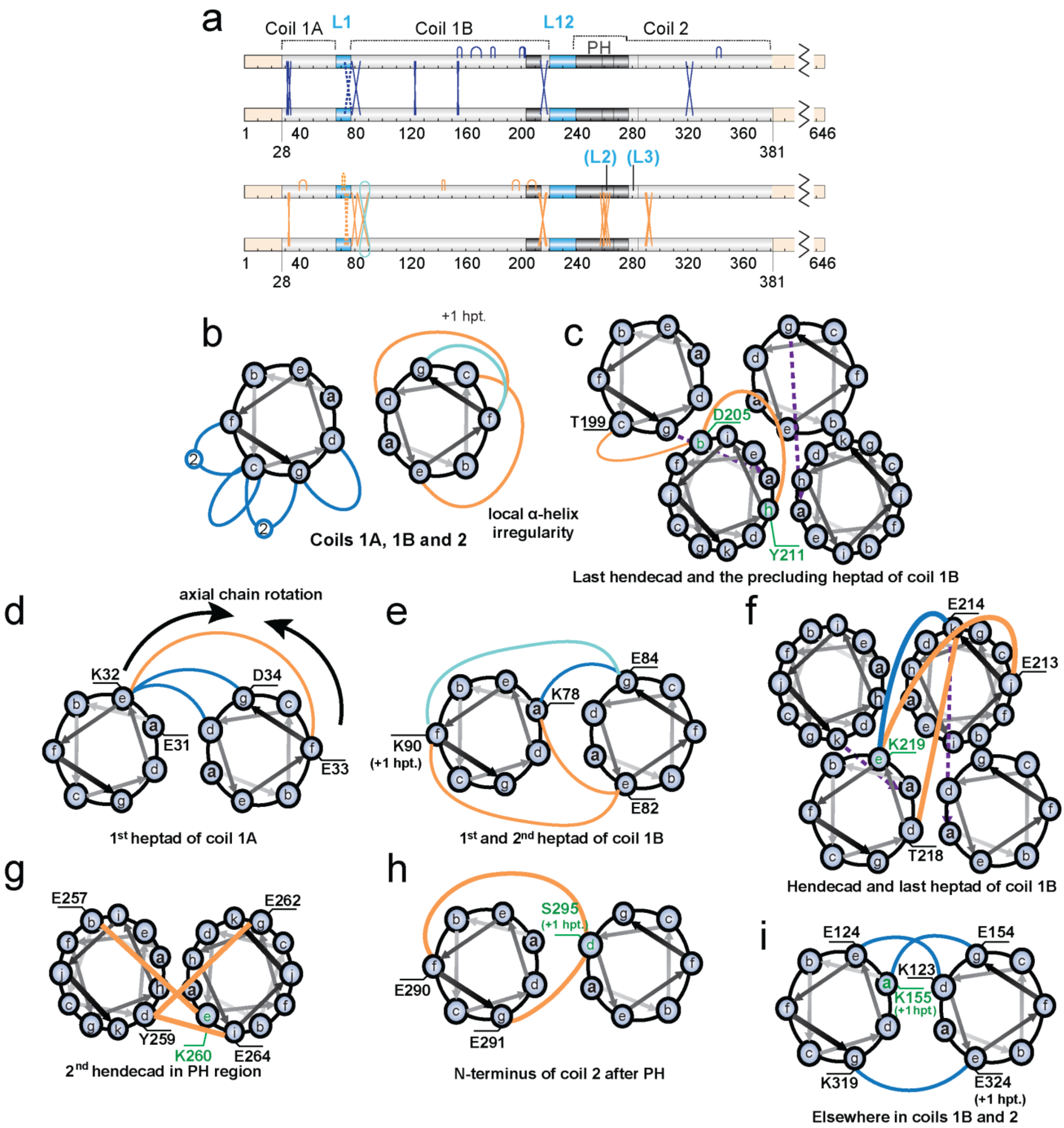

Supplementary Fig. 3. Cross-links in the coiled coil segments of the lamin A dimer rod. a A total of 31 cross-links were found in the lamin A dimer rod domain that satisfy classical coiled coil geometry: 11 intra-chain, 19 inter-chain cross-links (blue and orange lines both) and 1 cross-link occurring both ways (cyan lines). **b-i** Coiled coil wheel projections show predicted heptad and hendecad positions for residues in respectively intra-chain (***b,c***) and inter-chain (***d-i***) cross-links shown in (***a***)**.** Blue lines show cross-links with accessible side chains predicted to be within range of the EDC cross-linking agent. These are in compliance with the predicted α-helical coiled coil geometry in coils 1A, 1B and 2. Orange lines indicate cross-links between residues

with side chains inconsistent with such idealised static α-helical coiled coil backbone and potentially indicate irregularities in the lamin A coiled coil rod. However, most of these could be satisfied with an alternative axial chain rotation (example arrows for 1st heptad of coil 1A) in N-termini of coils 1A and 2, both termini of coil 1B and in PH. Such rotation is supported by 11 inter-chain cross-links and consistent with chain separations suggested for coil 1A and PH[1, 2] and/or irregularities accompanying hendecad-to-heptad transition regions in coil termini [3, 4, 5] (Supplementary text). A single intra-/inter-chain cross-link highlighted in cyan implies either axial chain rotation or local chain irregularity. Three cross-links were within the L1 region (dashed lines) and are not shown on wheel projections.

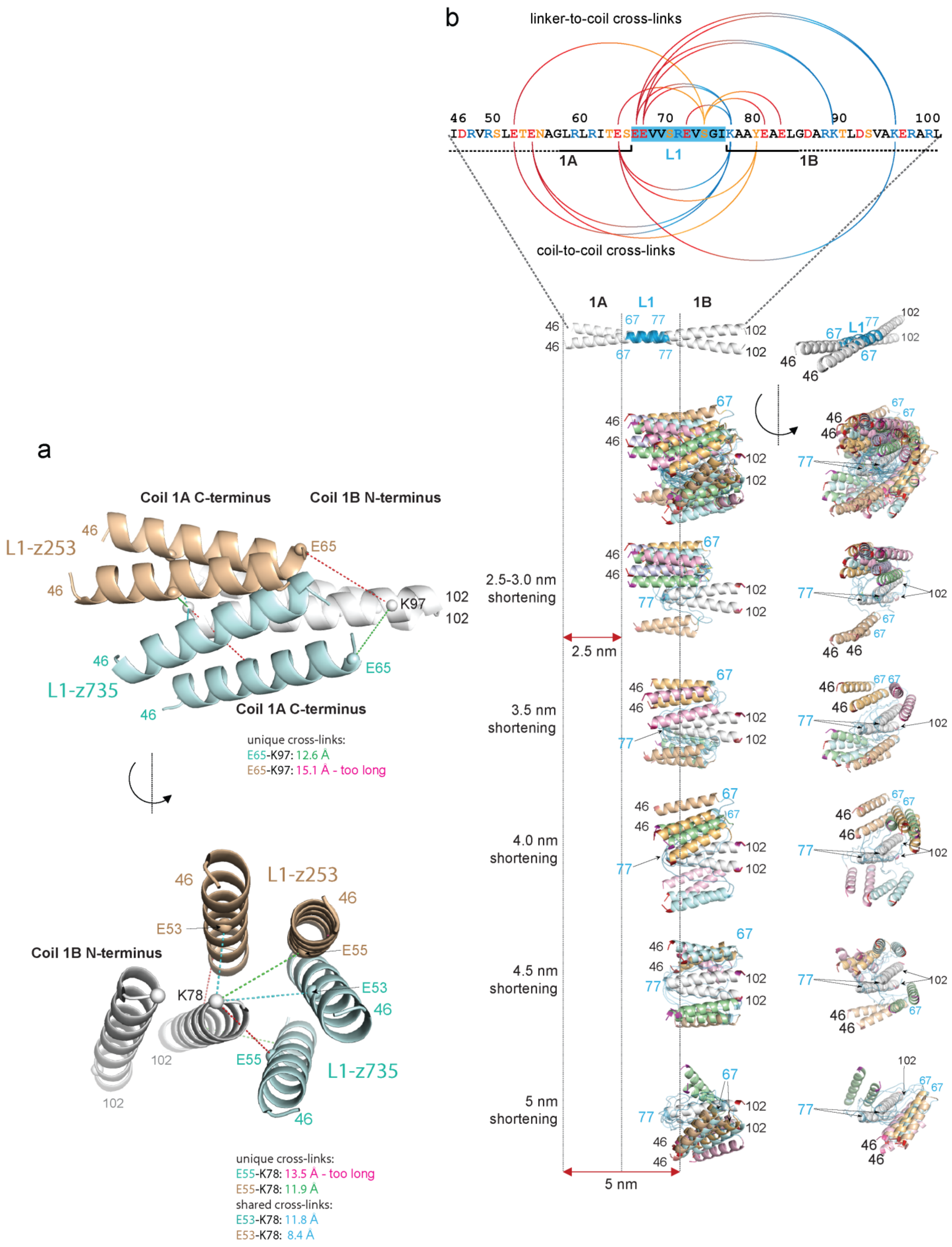

Supplementary Fig. 4. Lamin A linker L1 tandem stagger interactions and models. a Example of the two rod/L1 tandem stagger folding models with overlapping C- and N-termini of coils -1A and –1B respectively. Cross-link E65-K97 is satisfied in z735 model, but not in z253.

Cross-link E55-K78 on the other hand is only possible in z253. Both models satisfy an E53-K78 cross-link. Interface scores (I_sc) for models z735 and z253 were -6.70 and -6.73 respectively attesting to a relatively stable coiled coil termini docking in both cases. **b** A total of 221 stable (I_sc < -5.0) rod/L1 tandem stagger fold models were generated by Rosetta that satisfied polar and electrostatic interactions between charged (blue and red letters in the sequence) and polar (yellow letters) residues captured in cross-links (gradient lines). 5 out of 6 cross-links in 18 out of 24 intra/inter-chain variations were satisfied in these models. Example model sets show different degrees of overlap between coils -1A and -B that were dictated by different sub-sets of cross-links during modelling. Resulting rod shortening thus varies accordingly from 2.5 to 5.0 nm. Molecular modelling data for the cross-linked guided and un-guided Rosetta runs are available via Edinburgh DataShare (https://datashare.is.ed.ac.uk/handle/10283/3348) and its annotation in the Supplementary Data 7, 8 and 12.

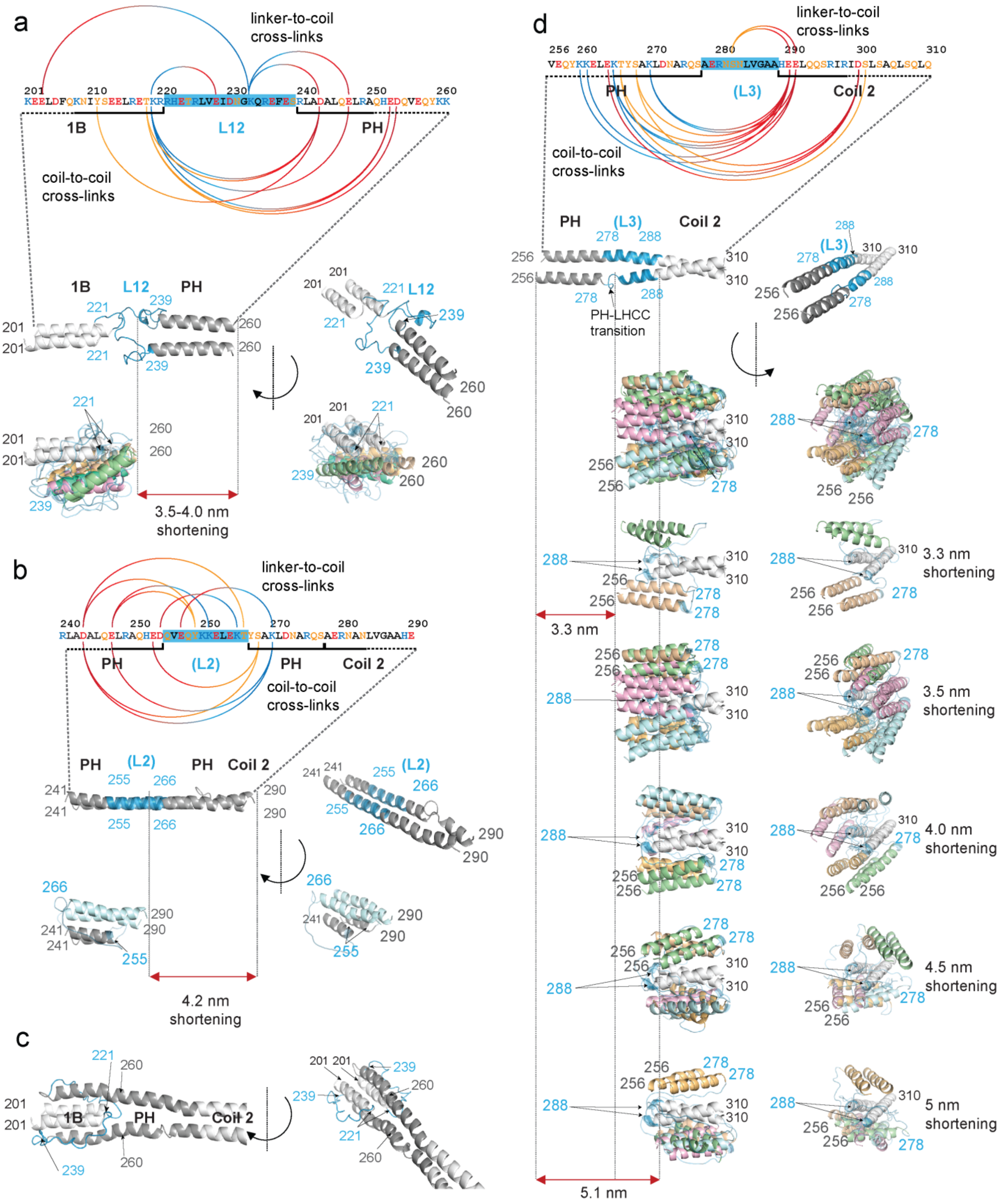

Supplementary Fig. 5. Lamin A linker L12, L2 and L3 tandem stagger interactions and models. a-c Modelling of rod tandem stagger folding in L12 (***a***) and L2 (***b***) yielded respectively only 4 stable interface models satisfying 3 out of 7 input Coil 1B-to-PH cross-links in 10 out of 20 possible intra/inter-chain variations and a single stable interface model satisfying 1 out of 4 input cross-links across linker L2. The degree of rod shortening observed varies between 3.5 and 4.0 nm in rod/L12 tandem stagger fold models and was 3.7 in the rod/L2 tandem stagger fold model. An alternative tandem stagger folding geometry for

these fragments may include an additional chain separation in coiled coil termini themselves during overlap as in the mock model shown in (***c***). **d** Modelling of the coiled coil termini overlap to satisfy cross-links around L3 yielded 60 models with relatively stable coil-to-coil interfaces satisfying 11 out of 12 coil-to-coil cross-links in 42 out of 48 possible intra/inter-chain variations. Degree of rod shortening from rod/L3 tandem stagger folding varied between 3.3 and 5.1 nm. Linker-to-coil and coil to-coil cross-links between charged (red and blue letters in sequences) and polar residues (orange letters) are shown for each tandem stagger folding region (gradient lines). Molecular modelling data for the cross-linked guided and un-guided Rosetta runs are available via Edinburgh DataShare (https://datashare.is.ed.ac.uk/handle/10283/3348) and its annotation in the Supplementary Data 7, 9-11 and 13-15.

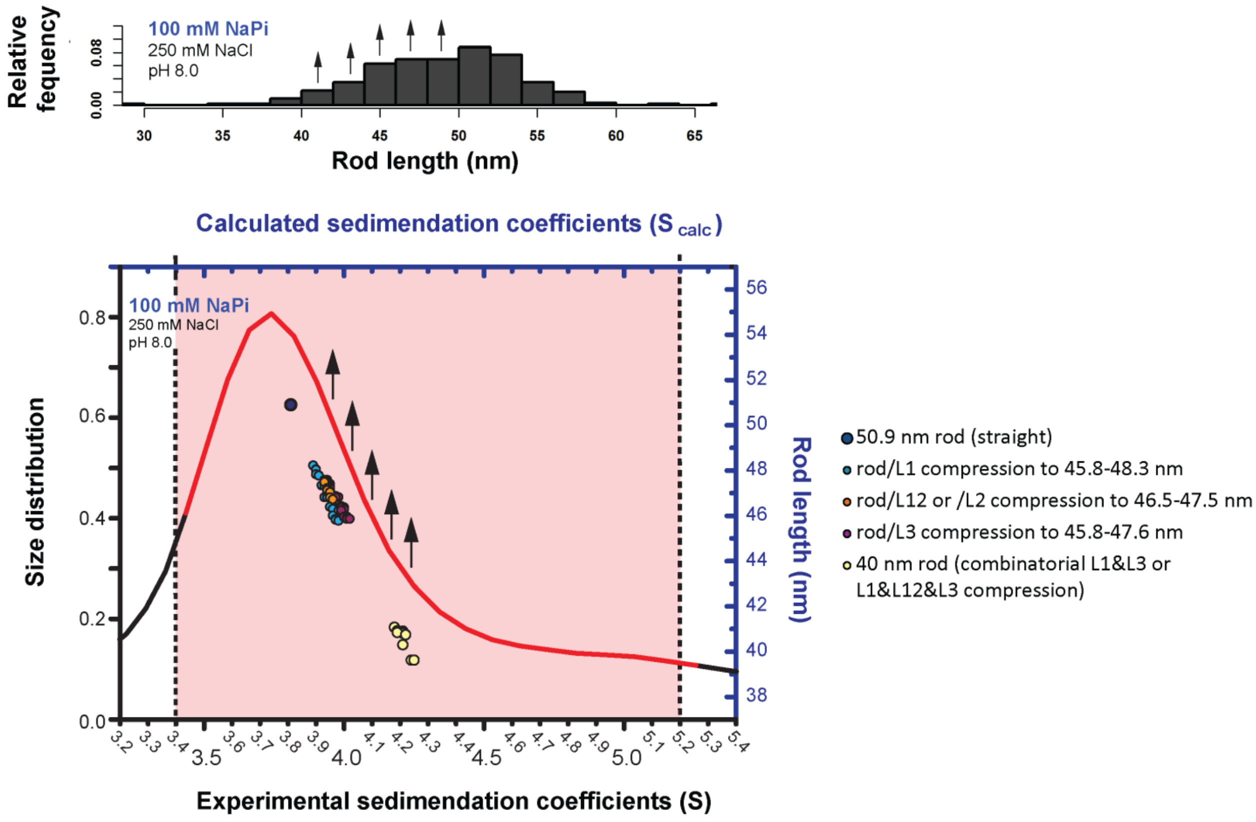

Supplementary Fig. 6. Modelled dimer rod shortening fits with analytical ultracentrifugation data. The un-cross-linked lamin A rod length distribution in phosphate buffer measured by rotary metal shadowing EM (top, from **Fig. 1c**) and analytical centrifugation curve for un-cross-linked lamin A in the same buffer (bottom, black axis set, from **Fig. 1d**). Hydrodynamic calculation of sedimentation coefficients was carried out for a set of lamin A dimer models in stages of tandem stagger folding compression fitting EM measurements. This yielded increased S-values for shorter dimers (bottom, dots, blue axis set) that are within integration peak limits of the experimentally observed analytical centrifugation curve (bottom, red curve). S-values were calculated for all of the obtained L12 and L2 tandem stagger fold models. For L1 and L3 five models were selected roughly for every 0.5 nm of the rod length decrement between that minimally and maximally observed in these model sets. SoMo source data is available in the Source Data file.

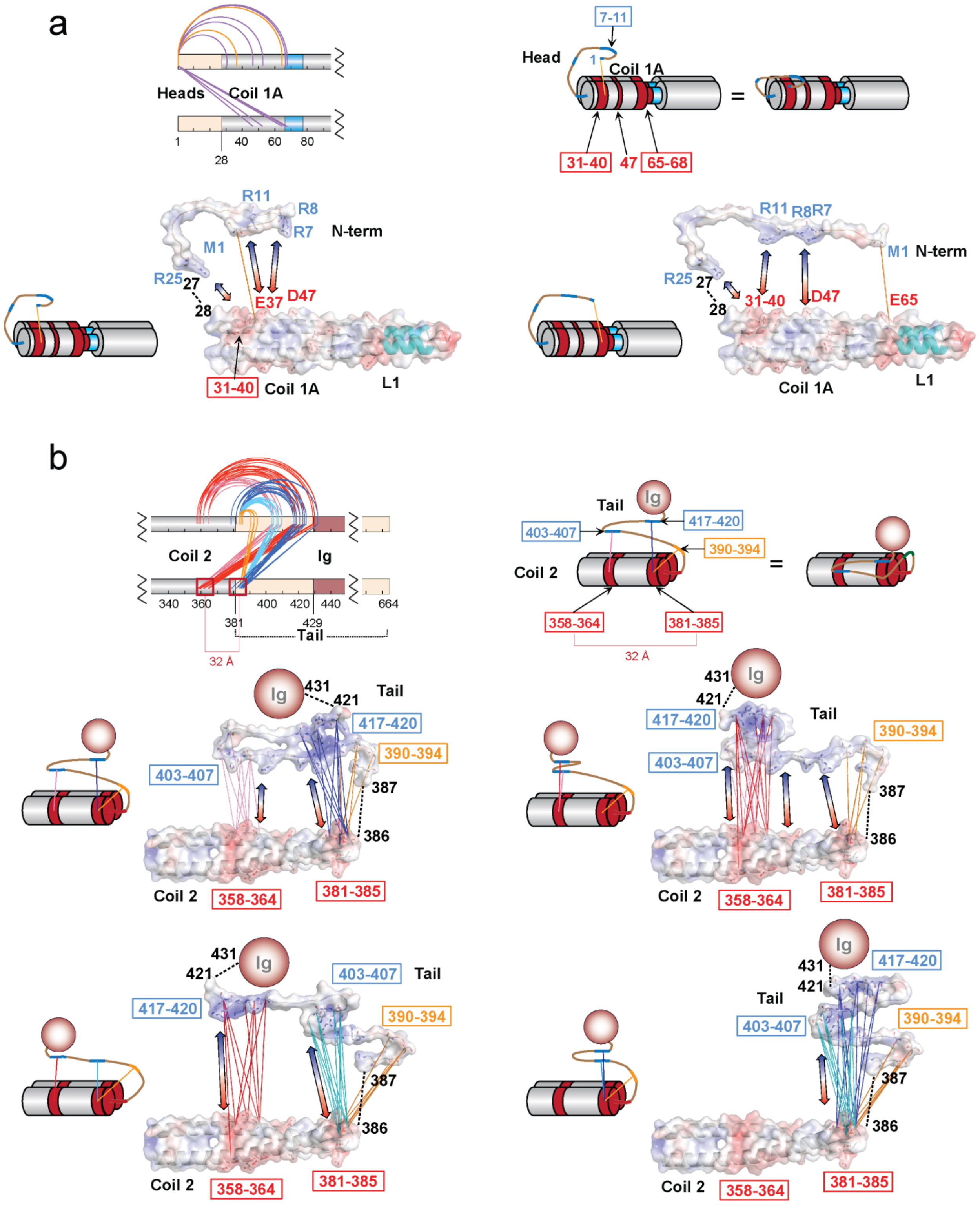

Supplementary Fig. 7. Dynamic head and tail folding onto the respective rod termini. Line schematics indicating cross-links are shown on the top, schematic "cylinder" models and conceptual structural models with electrostatic surface potential reconstructions shown on the bottom. Positively and negatively charged sites are shown in blue and red on schematics and electrostatic surface potential reconstructions (red-to-blue colouring– negative-to-positive

charges: -5 to +5 *kT*); electrostatic attractions supporting multiple fold conformations are indicated (gradient-filed lines). **a** A total of seven cross-links between N-terminal methionine and acidic residues along the coil 1A and L1 were identified. Example models show sufficient length of the head when aligned to satisfy cross-links on either end of coil 1A (highlighted in green) and favourable electrostatic interactions likely to support either conformation. **b** A total of 56 cross-links were identified to support the tail folding onto the rod C-terminus, the majority of which had two acidic sites – [358-364] and [381-385] – in coil 2 combinatorially cross-link to two positively charged sites in the tail – [403-407] and an NLS-containing site [417-420] (cross-link sets are coloured respectively red, pink, light and dark blue). Mitotic phosphorylation site [390-394] additionally cross-linked to [381-385] (green cross-links). As the two acidic coil 2 sites are too far away (32 Å) for any residue in the tail to cross-link to both at the same time, these cross-links likely capture multiple conformations of the tail folded onto the rod as shown on schematics and example models.

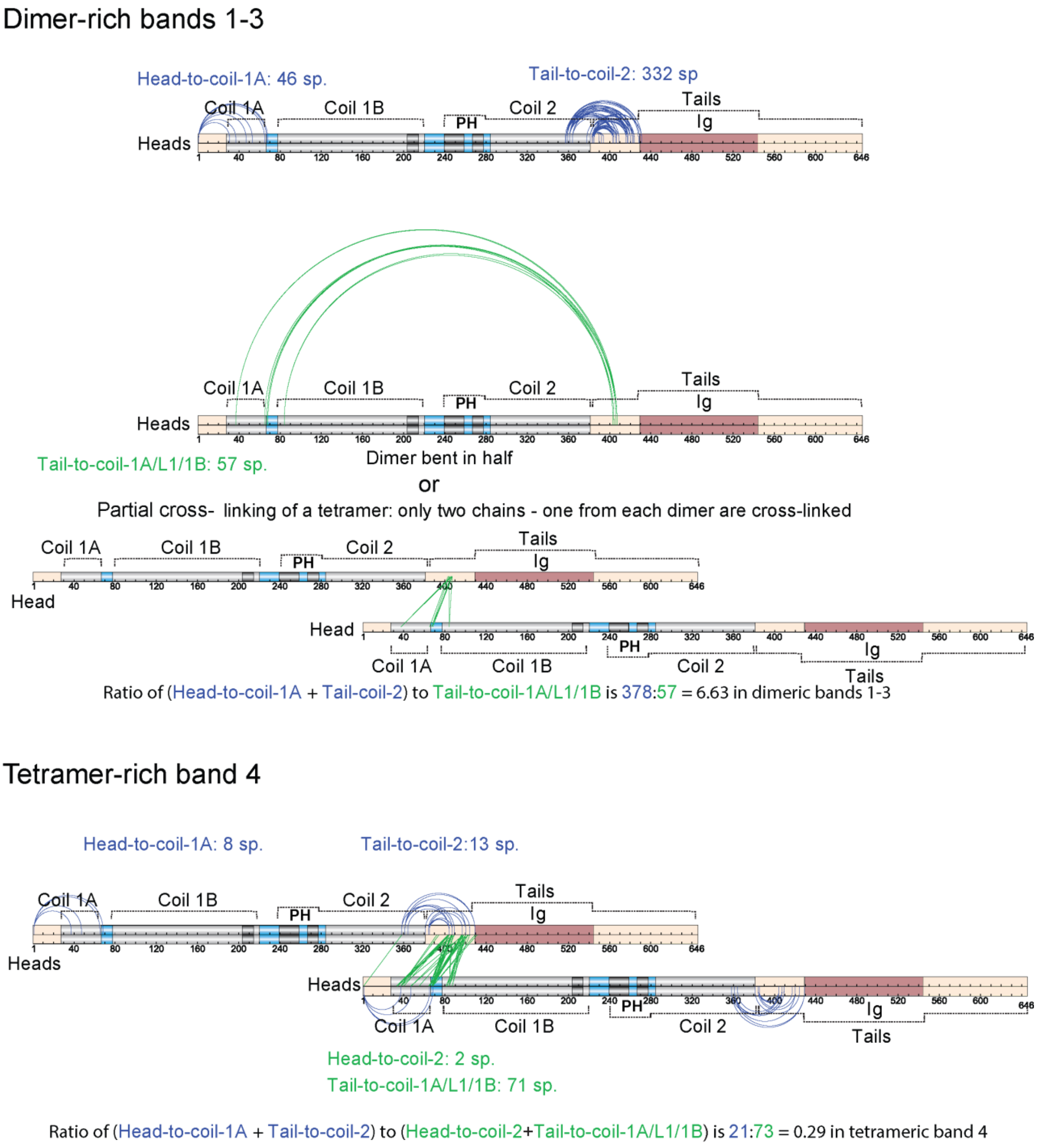

Supplementary Fig. 8. Head and tail interactions shift to inter-dimeric upon tetramerisation. Intra-dimeric Head-coil1A and Tail-coil 2 cross-links are prevalent (378 spectra) when compared to Head-coil 2 and Tail-coil1A/1B cross-links (57 spectra) in dimeric bands (relative ratio of 6.63). Note that Head-coil 2 cross-links are altogether absent in dimeric bands, while Tail-coil1A/1B cross-links may have arisen from either lamin A dimers bent in-half in L12 or from the products of incomplete cross-linking of tetramers where only two out of 4 chains were cross-linked by EDC. By contrast intra-dimeric Head-coil1A and Tail-coil 2 cross-links (21 spectra) are dominated by the inter-dimeric Head-coil 2 and Tail-coil 1A/1B cross-links

(73 spectra) in the tetrameric band (relative ratio of 0.29). For list of cross-links and spectra numbers see Supplementary Data 6.

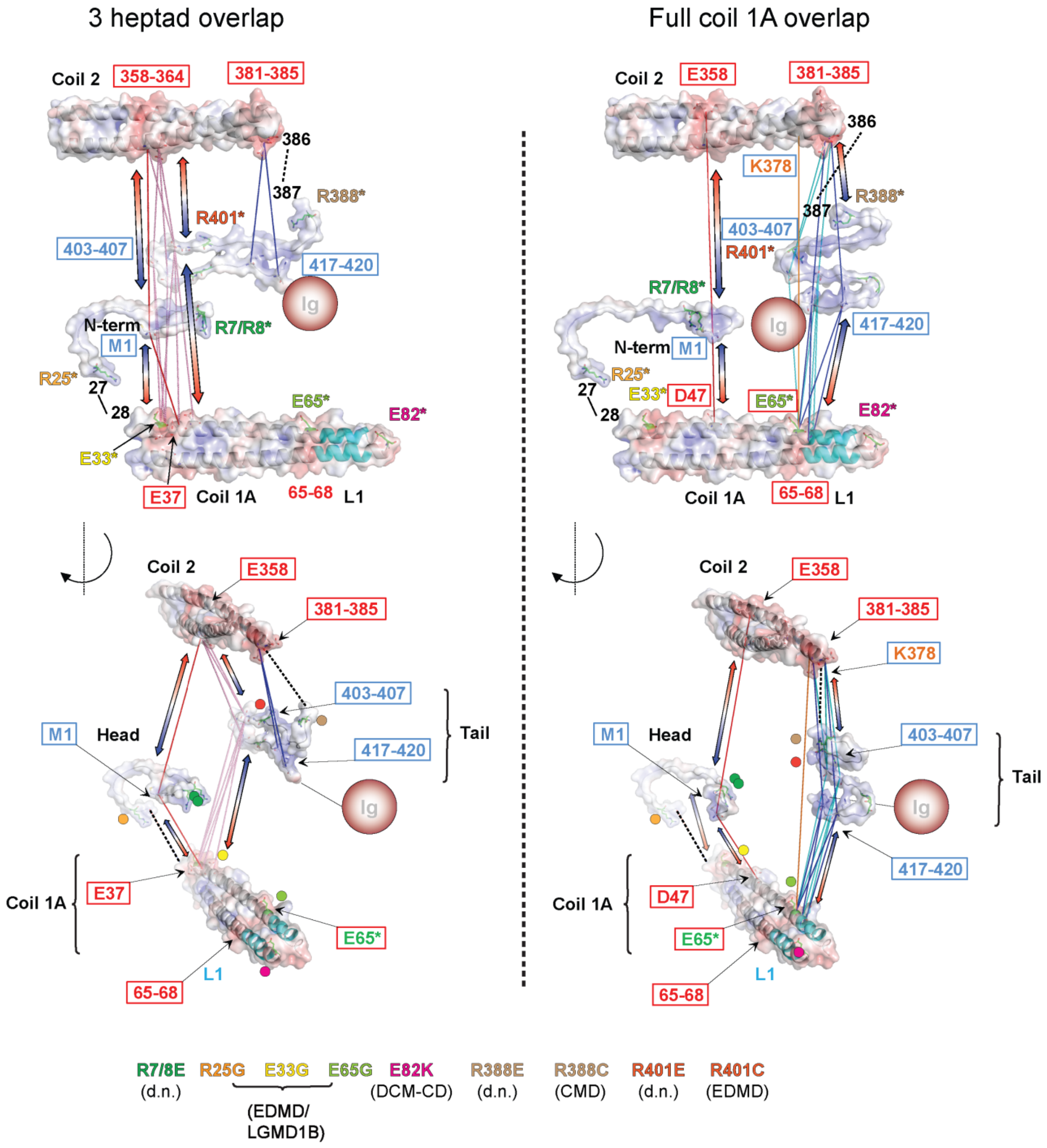

Supplementary Fig. 9. "Head-to-tail" interface assisted by unstructured head and tail regions. Side view (top) and 3D view (bottom) of the modelled projections of the lamin A tetrameric interface with the predicted 3 heptad overlap or a full coil 1A overlap, supported by the E65-K378 cross-link. Cross-linked residues/sites are boxed and cross-links are coloured accordingly. All models are overlaid with electrostatic surface potential reconstructions (red to blue surface colouring indicates negative to positive charges from -5 to +5 kT respectively) that suggest a favourable electrostatic attraction between head/tail and coil termini (gradient-filled

arrows). Several residue side chains are drawn in green and corresponding positions are marked with (*) or coloured circles. *de novo* and disease-associated mutations in these residues are listed at the bottom of the figure. These are potentially disruptive for the proposed electrostatically-driven head-to-tail interface and were studied for their effects on lamin A assembly *in vitro* (Figure 8f,g).

Supplementary Discussion

Fitting cross-linking data onto the basic lamin A dimer model: Cross-linking as a snapshot of conformational variety in a population of flexible protein molecules

General fitness of the prediction-based model and experimentally collected cross-links was assessed computationally using Xwalk [6] and manually in PyMol by measuring inter-atomic distances and by rotamer manipulation. As lamin A dimer has three major arch-domains (head, rod and tail) of which two are largely disordered (head and tail, excepting the Ig domain) the first emphasis in our analysis was on validation of the rod domain structure. First cross-links were assessed for compliance by distance: if cross-linked residues are within range of EDC cross-linker assuming 1.5 Å per residue in a coiled coil [7]. In this first instance the relative axial rotation of the α-helices in the coiled coil was ignored. Cross-links between residues in general range for EDC cross-linking were then each fitted onto the modelled structure and checked for compliance with α-helical and coiled coil geometry by Xwalk and manually: if the side chains of cross-linked residues are not obscured from each other by the α-helical coiled coil backbone and further if these are in contact or can be rotamer-ed to become so. Crucial for this analysis was the ability to distinguish predominantly intra-, inter- or otherwise cross-links occurring both ways as enabled by SILAC CLMS setup. Inter-chain cross-links carried information about register in the general chain alignment and further about the interface between to chains. Intra-chain cross-

links, on the other hand, carried information of local chain geometry and as well of the residue side chain accessibility in the context of the coiled coil α-helix. Cross-links occurring both as intra- and inter-chain, in a way, carried information about both.

Based on this analysis all cross-links were classified into one of the following categories: 1) cross-links that comply with all of the above criteria (between residues with side chain sufficiently proximal for EDC to cross-link, with un-obscured side chains) were deemed as in agreement with the predicted lamin A rod structure; 2) intra-chain cross-links between proximal residues with side chains obstructed by the α-helical coiled coil backbone and imply alternative local protein backbone geometry; 3) inter-chain cross-links that can be satisfied by relative axial chain rotation to turn cross-linked residue side chain towards each other; 4) cross-links between residues with side chains that cannot be in contact (non-proximal residue cross-links) and require extensive re-organisation of protein chain relative alignment or protein chain remodelling leading to spatial repositioning of protein domains.

Principally, cross-links agreeing and disagreeing with a particular local lamin A rod geometry (as described in the previous section) found within the same small regions of the rod were not necessarily deemed contradictory. This is because cross-linking reaction was deliberately not carried to a 100% efficiency (Figure 1f, lines c and X) for the fear of saturating protein surface interface and collapsing the protein structure by over-cross-linking. Thus identified cross-links do not have to originate from the very same dimer molecule. Further, in view of the inherent flexibility of lamin A dimers that can be easily observed by EM, we postulate that collected cross-linking data provides a snapshot of multiple protein conformations that existed in the population during cross-linking reaction. This notion is particularly crucial for analysis of irregularities in the rod domain and understanding the concept of alternative axial chain rotation possibly happening within the coiled coil termini (separately discussed in the following section); and the multitude of ”tandem stagger” models satisfying input cross-links on a “several-at-a-time” basis. The same principle applied during the analysis of the cross-links

between the head and the rod or the tail and the rod: incompatible cross-links in these regions were deemed as reflective of a dynamic nature of the electrostatically-driven interaction between the head/tail disordered regions and the rod termini, both in the free dimers and in the head-to-tail oligomers.

Cross-links capture anticipated irregularities in otherwise parallel in-register coiled coil lamin A rod domain

A total of 31 cross-links between proximal residues were found in the coiled coil segments of lamin A rod domain (Supplementary Figure 3a, Supplementary Data 5). Among these 8 out of 19 inter-chain cross-links and 6 out of 11 intra-chain cross-links (Supplementary Figure 3a top, blue lines) fit static model of lamin A based on prediction and crystal structures of homologous regions in vimentin cytoplasmic intermediate filament protein. Most notably these cross-links support canonical coiled coil core interface interactions between residues in heptad positions "a" or "e" and "d" or "g" (Supplementary Figure 3d-i, blue lines) in and at the termini of coils 1A, 1B and 2. This, however, leaves 11 inter-chain, 5 intra-chain (Supplementary Figure 3a top, orange lines) and one cross-link (same, cyan lines) happening both ways that mostly cluster in coiled coil segment termini and do not outright support the static model. Instead these reveal some anticipated irregularities in the chain geometry and alternative chain packing of coiled coil segment termini and attest to the likely flexibility of these.

In coil 1A N-terminus residue K32 in heptad position "e" was found to canonically interact with residues E31 and D34 in positions "d" and "g" respectively, but also with residue E33 in heptad position (f). In the static coiled coil model K32 and E33 would be obscured from each other by the coiled coil α-helical backbone, thus such interaction would necessitate either a non-coiled coil α-helical chain geometry, or a chain separation and axial chain rotation (Supplementary Figure 3d) that could bring these residues to face each other. Such low stability

of the coiled coil and chain separation is indeed noted for coil 1A in protein fragment crystal structures [7, 8] of vimentin and is attributed to a specific conserved tyrosine residue in core heptad position “d” Y117 in vimentin and homologous Y45 in lamin A [1, 8]. Our data thus further validates these observations for full length lamin A and suggests that coil 1A termini could temporarily associate in several different conformations.

We further find evidence for similar chain separation/axial twisting in parallel hendecad (PH) region of coil 2 where three inter-chain cross-links (E257(b)-K260(e), Y259(d)-E262(g) and Y259(d)-E264(i), hendecad positions in brackets) all contradict the assigned core hendecad coiled coil interface (Supplementary Figure 3g). This data again supports previous structural evidence of instability in PH region where two α-helices only form a hendecad coiled coil dimer if additionally stabilised at the N-terminus in fragment crystal structures [2, 5]. We suspect that, similarly to coil 1A, conserved tyrosine residues Y259 and Y267 in core hendecad positions “d” and “a” respectively contribute to the PH instability in full length lamin A dimers.

Additional coiled coil chain packing irregularities were observed at both termini of coil 1B and in heptads following the PH region in coil 2. While less anticipated, these however fit some of the theoretical and experimental observations published in the past. Linker L1 is thought α-helical in lamins but interrupts the continuous heptad pattern from coil 1A to 1B [9]. Such interruptions, or stutters are thought to cause compensatory local chain irregularities [3] that can explain similar to coil 1A contradictory cross-linking patterns in the first two heptads of coil 1B (Supplementary Figure 3e) and linker L1. The extend of such irregularity is hard to estimate, but notably core heptad position “d” in the first heptad of coil 1B is again occupied by a tyrosine reside (Y81) which can be causing a chain separation and axial chain rotation similar to one in coil-1A. Similar argument can be made for the C-terminus of coil 1B and coil 2 where contradictory intra- and inter-chain cross-links (Supplementary Figure 3c,f,h) can arise from chain irregularities caused by hendecad-to-heptad transitions [4] experimentally observed in

vimentin [5, 8, 10] or local chain separation and twisting (for coil 1B C-terminus, supported by tyrosine Y211 in core hendecad position "h").

Notably, all of the cross-links discussed above are between residues within range of EDC cross-linking in the predicted parallel and in-register coiled coil model of the rod domain and thus support it. A number of cross-links between residues with side chain to far apart in such model to be cross-linked were also found (Figure 6b, Supplementary Data 5). One tempting explanation for such apparent abundance of these cross-links within lamin A rod domain may lay in the possibility of the relative chain sliding, or "out-of-register" chain alignment. Indeed such alignment could satisfy a number of cross-links across linker regions. However among all the cross-links between the non-proximal residues in the rod only three cross-links are predominantly inter-chain and can be explained by L12 Z-folding (T218(d)-D243(j)), or by the L3 Z-folding or a smaller irregularity in α-helical geometry caused by parallel hendecad-to-heptad coiled coil transition following the PH region of coil 2 (S282(e)-E290(f) and S282(e)-E291(g)). Remaining cross-links, with the exception of one, are all either intra-chain (and thus cannot be satisfied by chain sliding) or occur both ways as intra- and inter-chain. While an inter-chain cross-link for a particular pair of residues may be explained through chain sliding, the same cross-link occurring as an intra-chain would still not be possible without additional chain refolding (i.e. compression or bending over). Given the general "normal" rod architecture as observed by EM and the abundance of cross-links that occur both ways it seems unlikely that the population would include two alternative "out-of-register" and "compressed" dimer populations for every such cross-link. The only exception from this is a predominantly inter-chain cross-link E173(e)-S268(b) which is one of the several non-proximal residue cross-links from coil 1B to coil 2 (all intra-chain or both-ways cross-links) that probably rose from dimer folding in-half in L12 as these cross-links are between residues lying too far apart to be accommodated by tandem stagger architecture (Supplementary Data 5, "Other cross-links").

Putative linkers (L2) and (L3) in the coil-2

While currently accepted model of the intermediate filament protein rod domain organisation implies only two linkers – linker L1 between coils 1A and 1B and L12 between coils 1B and 2 – this has not always been the case. Flanked by two bulky tyrosine residues Y259 and Y267 linker L2 in the middle of the PH region of coil 2 was historically thought to separate coil 2 into two coiled coil segments – coil 2A and coil 2B [9]. In this study the second full hendecad of PH containing this linker (residues 256-266) was thus termed as putative linker (L2) and investigated for ability to fold to accommodate tandem stagger and satisfy non-proximal residue cross-links in the PH region. Molecular modelling however indicated that a stable interface between two halves of PH region is unlikely without a further chain refolding or a noticeable kinking of the rod, leaving doubts as to the nature of this linker. The existence of the forth linker was never proposed, although according to an older prediction from Gerace lab coil 2A may contain 6 full heptads rather than two and a half [11] and residues 283-288 in the first two heptads downstream of the PH were annotated as linker (L3). Notably however, in the current model this region coincides with the parallel hendecad-to-heptad transition after the PH region of coil-2. Thus in this study we annotated the first two heptads of coil 2 after the PH (residues 278-288) as a putative linker (L3). We speculate that the unique sequence features of this region such as 3 flexible, sterically un-hindered alanine residues at both ends and non-α-helix-friendly asparagine and glycine residues N281, N283 and G286 [12] may be contributing to the flexibility necessary for the (L3) folding and formation of coiled coil tandem stagger.

Supplementary Tables

Supplementary Table 1

Ex vivo rat liver cross-linking						
Region	Residue - Residue	Region	Spectral count	Inter-molecular *in vivo*: overlapping peptides	*Intra-/inter-chain* in vitro: *SILAC*	Annotation
Head	S12 - E33	1A	1			Head folding onto the rod
1A	E31(d) - K32(e)	1A	4	yes	inter-chain	classic heptad coiled coil d-e interaction
1A	E65(c) - K97(f)	1B	1		both	1A/1B termini tandem stagger
L1	E68 - S94(c)	1B	1			L1 folding onto coil 1B: 1A/1B termini tandem stagger
L1	E68 - K97(f)	1B	2		both	L1 folding onto coil 1B: 1A/1B termini tandem stagger
L1	S71 - E73	L1	1		intrachain	
1B	S143(c) - E145(e)	1B	2	yes	intra-chain	Inter-molecular *ex vivo*, but intra-chain *in vitro*
1B	D192(c) - S239	L12	1			L12 folding onto coil 1B: 1B/PH termini tandem stagger
1B	E194(e) - T199(c)	1B	1			
1B	T199(c) - E202(f)	1B	3		intra-chain	
L12	S239 - D243(j)	PH	1			
PH	E247(c) - K378(f)	2	15		inter-dimeric	Inter-dimeric *in vitro*. Lateral anti-parallel half-staggered tetramerisation via coils 2
PH	K270(d) - E361(c)	2	1		inter-dimeric	Inter-dimeric *in vitro*. Lateral anti-parallel half-staggered tetramerisation via coils 2
(2-α.hel.ext)	E385 - S407	Tail-N	1		intra-chain	Tail folding onto the rod
Tail-N	E425 - S428	Tail-N	4		intra-chain	

***Ex vivo* Nuclear Envelope cross-linking results.** In blue and magenta are cross-links previously encountered *in vitro*, in green de novo cross-links. Given are domain assignment, residue position and type, spectral counts and annotation.

Supplementary Table 2

Construct	Primer name	Primer sequence
Untagged- lamin A-ΔC18	Unt-LA-C1389T-SDM-fwd	5'CAATGAGGACCAGTCTATGGGCAATTGGCAGA-3
	Unt-LA-C1389T-SDM-rev	5'-TCTGCCAATTGCCCATAGACTGGTCCTCATTGG-3'

	Unt-LA-NcoI-fwd	5'-ACGGGATCCCATGGAGACCCCGTCCCAGCGGC-3'
	Unt-LA-BamHI-rev	5'-GCGGATCCTCAGTAGGAGCGGGTGACCAGATTGTC-3'
6xHis-lamin A-ΔC18	6xHis-LA-BamHI-fwd	5’-ACGGGATCCGATGGAGACCCCGTCCCAGCG-3’
	6xHis-LA-XhoI-rev	5’-GCCTCGAGTCAGTAGGAGCGGGTGACCAGATTGTC-3’
Untagged-lamin A-ΔC18-R7/8E	Unt-LA-R7/8E-SDM-fwd	5'-CCCCGCTGCGGGTGGCTTCTTCCTGGGACGGGGTCTCC-3'
	Unt-LA- R7/8E -SDM-rev	5'-GGAGACCCCGTCCCAGGAAGAAGCCACCCGCAGCGGGG-3'
Untagged-lamin A-ΔC18-R25G	Unt-LA-R25G-SDM-fwd	5'-CCGGGTGATGCCGGTGGGCGACA-3'
	Unt-LA-R25G-SDM-rev	5'-TGTCGCCCACCGGCATCACCCGG-3'
Untagged-lamin A-ΔC18-E33G	Unt-LA-E33G-SDM-fwd	5'-AGCTCCTGCAGGTCGCCCTTCTCCTGCAGC-3'
	Unt-LA-E33G-SDM-rev	5'-GCTGCAGGAGAAGGGCGACCTGCAGGAGCT-3'
Untagged-lamin A-ΔC18-E65G	Unt-LA-E65G-SDM-fwd	5'-TGACCACCTCTTCAGAACCGGTGATGCGAAGGCG-3'
	Unt-LA-E65G-SDM-rev	5'-CGCCTTCGCATCACCGGTTCTGAAGAGGTGGTCA-3'
Untagged-lamin A-ΔC18-E82K	Unt-LA-E82K-SDM-fwd	5'-CGAGCTCGCCTTGTAGGCGGCCTT-3'
	Unt-LA-E82K-SDM-rev	5'-AAGGCCGCCTACAAGGCCGAGCTCG-3'
Untagged-lamin A-ΔC18-R388C	Unt-LA-R388C-SDM-fwd	5'-GGGGGACAGGCATAGCCTCTCCTCCT-3'
	Unt-LA-R388C-SDM-rev	5'-AGGAGGAGAGGCTATGCCTGTCCCCC-3'
Untagged-lamin A-ΔC18-R388E	Unt-LA-R388E-SDM-fwd	5'-AGGGCTGGGGGACAGTTCTAGCCTCTCCTCCTCG-3'
	Unt-LA-R388E-SDM-rev	5'-CGAGGAGGAGAGGCTAGAACTGTCCCCCAGCCCT-3'
Untagged-lamin A-ΔC18-R401C	Unt-LA-R401C-SDM-fwd	5'-GCGCAGCCGTGGCTGCGCTTCCTCTCACT-3'
	Unt-LA-R401C-SDM-rev	5'-AGTGAGAGGAAGCGCAGCCACGGCTGCGC-3'

Untagged-lamin A-ΔC18-R401E	Unt-LA-R401E-SDM-fwd	5'-TGAGTGAGAGGAAGCTTCGCCACGGCTGCGCTG-3'
	Unt-LA-R401E-SDM-rev	5'-CAGCGCAGCCGTGGCGAAGCTTCCTCTCACTCA-3'

Cloning Primers. Primers used for removing and internal NcoI restriction site from human lamin A sequence (via mutation at position 1389); for cloning of un-tagged and 6xHis-tagged human lamin A sequence into pET28b vector for bacterial expression; site-directed mutagenesis primers for introducing *de novo* and disease associated mutations.

Supplementary Note 1

Rosetta global docking command:

```
rosetta_bin_linux_2017.08.59291_bundle/main/source/bin/docking_protocol.static.linuxgccrelease @flag_global_docking
```

Contents of flag_global_docking to generate 100,000 models:

```
-in:file:s in.pdb
-unboundrot in.pdb
-nstruct 100000
-partners AB_CD
-spin
-randomize1
-randomize2
-ex1
-ex2aro
-docking:no_filters
-constraints:cst_file rosetta_crosslinks.txt
-out:overwrite
```

This was also run without the constraint file for coil 1A/1B overlap docking.

X-walk SAS distance calculation command:

```
java -Xmx1024m -cp xwalk/bin Xwalk -infile rosetta_out.pdb -dist xwalk_crosslinks.txt -out rosetta_out.pdb.txt -max 13 –xSC –space 0.5 > rosetta_out.out
```

Here initial maximal distance for SAS was set to 13 Å and results were further filtered for compliance with an individual maximal distance cut-off for each type of cross-link: GLU-LYS 12.2 Å; GLU-SER 8.3 Å; GLU-TYR 12.3 Å; GLU-THR 8.3 Å; ASP-LYS 11 Å; ASP-SER 7.1 Å; ASP-TYR 11.1 Å; ASP-THR 7.1 Å. Rotamer freedom for cross-linked residues was allowed (-xSC). Manual validation was additionally employed for cross-links with SAS close to maximal distance or less than 1 Å longer than maximal distance; and for cross-links with tyrosine residues as these have relatively rigid side chains with only $\chi1$ and $\chi2$ torsion angles providing rotamer freedom.

Rosetta local refinement command:

```
-in:file:s rosetta_global_out.pdb
-nstruct 1
-docking_local_refine
-use_input_sc
-ex1
-ex2aro
-out:file:fullatom
-out:path:all output_files_local_refine
-out:suffix _rosetta_local_refine
```

MODELLER linker reconstruction commands and input files:

```
from modeller import *
from modeller.automodel import *    # Load the automodel class

log.verbose()
```

```
env = environ()

# directories for input atom files
env.io.atom_files_directory = ['.', '../atom_files']

class MyModel(dopehr_loopmodel):
    def special_patches(self, aln):
        self.rename_segments(segment_ids=('A', 'B'), renumber_residues=($chainstart, $chainstart))
    def select_atoms(self):
        return selection(self.residue_range('$gapstart:A', '$gapend:A'),
                         self.residue_range('$gapstart:B', '$gapend:B'))

a = MyModel(env, alnfile = 'Frag.ali',
            knowns = 'Known', sequence = 'model_to_fix')

a.loop.starting_model = 1
a.loop.ending_model   = 9
a.loop.md_level       = refine.slow_large

a.make()
```

Modeller alignment files:

L1:

```
>P1;Known_L1
structureX:rosetta_local_out:28:A:102:D:x:x:x:x

RLQEKEDLQELNDRLAVYIDRVRSLETENAGLRLRITES-----------KAAYEAELGDARKTLDSVAKERARL/

RLQEKEDLQELNDRLAVYIDRVRSLETENAGLRLRITES-----------KAAYEAELGDARKTLDSVAKERARL*

>P1;model_to_fix
```

sequence:::::::::

RLQEKEDLQELNDRLAVYIDRVRSLETENAGLRLRITESEEVVSREVSGIKAAYEAELGDARKTLDS VAKERARL/

RLQEKEDLQELNDRLAVYIDRVRSLETENAGLRLRITESEEVVSREVSGIKAAYEAELGDARKTLDS VAKERARL*

L12:
>P1;Known_L12

structureX:rosetta_local_out:201:A:260:D:x:x:x:x

KEELDFQKNIYSEELRETK--------------------RLADALQELRAQHEDQVEQYK/
KEELDFQKNIYSEELRETK--------------------RLADALQELRAQHEDQVEQYK*

>P1;model_to_fix

sequence:::::::::

KEELDFQKNIYSEELRETKRRHETRLVEIDNGKQREFESRLADALQELRAQHEDQVEQYK/
KEELDFQKNIYSEELRETKRRHETRLVEIDNGKQREFESRLADALQELRAQHEDQVEQYK*

YSAKLDNARQSAERNSNLVGAAHEE*

L2:
>P1;Known_L2

structureX:rosetta_local_out:241:A:291:D:x:x:x:x

LADALQELRAQHED------------YSAKLDNARQSAERNSNLVGAAHEE/
LADALQELRAQHED------------YSAKLDNARQSAERNSNLVGAAHEE*

>P1;model_to_fix

sequence:::::::::

LADALQELRAQHEDQVEQYKKELEKTYSAKLDNARQSAERNSNLVGAAHEE/
LADALQELRAQHEDQVEQYKKELEKTYSAKLDNARQSAERNSNLVGAAHEE*

L3:
>P1;Known_L3

structureX:run rosetta_local_out:256:A:310:D:x:x:x:x

VEQYKKELEKTYSAKLDNARQ------------HEELQQSRIRIDSLSAQLSQLQ/

VEQYKKELEKTYSAKLDNARQ------------HEELQQSRIRIDSLSAQLSQLQ*

>P1;model_to_fix

sequence:::::::::

VEQYKKELEKTYSAKLDNARQSAERNSNLVGAAHEELQQSRIRIDSLSAQLSQLQ/

VEQYKKELEKTYSAKLDNARQSAERNSNLVGAAHEELQQSRIRIDSLSAQLSQLQ*

Supplementary References

1. Meier M*, et al.* Vimentin coil 1A-A molecular switch involved in the initiation of filament elongation. *J. Mol. Biol.* **390**, 245-261 (2009).

2. Nicolet S, Herrmann H, Aebi U, Strelkov SV. Atomic structure of vimentin coil 2. *J. Struct. Biol.* **170**, 369-376 (2010).

3. Strelkov SV, Burkhard P. Analysis of alpha-helical coiled coils with the program TWISTER reveals a structural mechanism for stutter compensation. *J. Struct. Biol.* **137**, 54-64 (2002).

4. Parry DA. Hendecad repeat in segment 2A and linker L2 of intermediate filament chains implies the possibility of a right-handed coiled-coil structure. *J. Struct. Biol.* **155**, 370-374 (2006).

5. Chernyatina AA, Strelkov SV. Stabilization of vimentin coil2 fragment via an engineered disulfide. *J. Struct. Biol.* **177**, 46-53 (2012).

6. Kahraman A, Malmstrom L, Aebersold R. Xwalk: computing and visualizing distances in cross-linking experiments. *Bioinformatics* **27**, 2163-2164 (2011).

7. Strelkov SV*, et al.* Conserved segments 1A and 2B of the intermediate filament dimer: their atomic structures and role in filament assembly. *EMBO J* **21**, 1255-1266 (2002).

8. Chernyatina AA, Nicolet S, Aebi U, Herrmann H, Strelkov SV. Atomic structure of the vimentin central alpha-helical domain and its implications for intermediate filament assembly. *Proc. Natl. Acad. Sci. U S A* **109**, 13620-13625 (2012).

9. Parry DA. Microdissection of the sequence and structure of intermediate filament chains. *Adv. Protein. Chem.* **70**, 113-142 (2005).

10. Aziz A*, et al.* The structure of vimentin linker 1 and rod 1B domains characterized by site-directed spin-labeling electron paramagnetic resonance (SDSL-EPR) and X-ray crystallography. *J. Biol. Chem.* **287**, 28349-28361 (2012).

11. Schirmer EC, Gerace L. The stability of the nuclear lamina polymer changes with the composition of lamin subtypes according to their individual binding strengths. *J. Biol. Chem.* **279**, 42811-42817 (2004).

12. Pace CN, Scholtz JM. A helix propensity scale based on experimental studies of peptides and proteins. *Biophys. J.* **75**, 422-427 (1998).
