# Supplementary material for: Lamin A molecular compression and sliding as mechanisms behind nucleoskeleton elasticity
Source: Nat Commun. 2019 Jul 11;10:3056. doi: 10.1038/s41467-019-11063-6 (PMC6624373; doi:10.1038/s41467-019-11063-6)
Supplement: Supplementary file 3 — Description of Additional Supplementary Files [file 41467_2019_11063_MOESM3_ESM.docx]

**Description of Supplementary Files**

**File Name:** Supplementary Data 1

**Description:** *Skyline XiC quantification of mono-, 1st- and 2nd isotopic peaks for a subset of spectra from HIDm X experiment*. Given among others are: spectra origin (experiment/band/SCX fraction); spectra number; supported cross-link; peak type for light-light (LL), heavy-light (HL), light-heavy(LH) and heavy-heavy (HH) cross-linked peptide pairs: mono- (m), 1st- (1) and 2nd isotopic peaks; pertinent linearised modified sequence with mass modifications in [ ], E[-131.1] stands for the cross-link, [+1] or [+9] (for heavy lysines) denote crosslinked residues; pertinent expected product Mz and Mass Errors; peaks within the same ion cluster family (same spectra) with overlapping masses are highlighted; Single peak areas and calculated (HL+LH)/(LL+HL+LH+HH) ratios for each spectra after factoring overlapping peaks.

**File Name:** Supplementary Data 2

**Description:** *Skyline XiC quantification of mono-, 1st- and 2nd isotopic peaks for a subset of spectra from H/hIDm X experiment*. Given among others are: spectra origin (experiment/band/SCX fraction); spectra number; supported cross-link; peak type for light-light (LL), heavy-light (HL), light-heavy(LH) and heavy-heavy (HH) cross-linked peptide pairs: mono- (m), 1st- (1) and 2nd isotopic peaks; pertinent linearised modified sequence with mass modifications in [ ], E[-131.1] stands for the cross-link, [+1] or [+9] (for heavy lysines) denote crosslinked residues; pertinent expected product Mz and Mass Errors; peaks within the same ion cluster family (same spectra) with overlapping masses are highlighted; Single peak areas and calculated (HL+LH)/(LL+HL+LH+HH) ratios for each spectra after factoring overlapping peaks.

**File Name:** Supplementary Data 3

**Description:** *Comparison of (HL+LH)/(LL+HL+LH+HH) ratios for a subset of spectra calculated using Skyline XiC areas or Xcalibur XiC average peak intensities.* Respective ratios are calculated using Skyline XiC areas (Supplementary Data 2, 3) or average peak intensities across XiCs recorded in Xcalibur (data given). With the exception of MS1 spectra with contaminant ions or otherwise poorly resolved isotopic clusters, Skyline and Xcalibur ratios closely match and are within 6% of each other for 95% of measurements.

**File Name:** Supplementary Data 4

**Description:** *List of all cross-links overlapping between HIDm X and H/hIDm X experiments in dimerrich bands 1-3; and all cross-links found in tetramer-rich band 4 of HIDm X experiment (Fig. 2 & 3).* Given are cross-linked residue positions, residue types, lamin A domain allocation and heptad/hendecad positions where applicable. Spectral counts are given. Given (HL+LH)/(LL+HL+LH+HH) ratios were calculated in Thermo Xcalibur. Corresponding derived frequencies of cross-link occurrence as inter-dimeric, inter-chain and intra-chain are plotted in Fig. 4. All dimeric cross-links are shown in Fig. 5 b and c.

**File Name:** Supplementary Data 5

**Description:** List of all cross-links within the rod domain in dimeric bands 1-3 (Fig. 5, 6). Given are cross-linked residue position, residue type, lamin A domain allocation, heptad/hendecad position where applicable as well as frequency of occurrence as inter-dimeric and intra-/inter-chain and a brief annotation.

**File Name:** Supplementary Data 6

**Description:** List of all cross-links involving at least one residue outside the rod domain in dimeric bands 1-3 and tetrameric band 4 (Fig. 5, 8). Given are cross-linked residue position, residue type, lamin A domain allocation, heptad/hendecad position where applicable as well as frequency of occurrence as inter-dimeric and intra-/inter-chain and a brief annotation.

**File Name:** Supplementary Data 7

**Description:** *Summary of cross-link-guided Rosetta molecular modelling runs.* Given are input crosslinks used for each of the suspect tandem staggers (residue type, number and chain), total number of models in which such a cross-link was satisfied, best and average Interface scores (I_sc), minimal, maximal and average rod shortening. Values are highlighted in red when a cross-link was only satisfied in symmetrical models: i.e. E65-K78 intra-chain cross-link was satisfied in a single model, not from chain A to chain A, but from chain B to chain B in the theoretically symmetrical lamin A homodimer.

**File Name:** Supplementary Data 8

**Description:** *List of tandem stagger models from cross-link guided Rosetta molecular modelling satisfying cross-links across linker L1.* Given are model names, number of satisfied cross-links, satisfied crosslinks, Interface score (I_sc), angle between the docked dimeric fragments and rod shortening conveyed by each model.

**File Name:** Supplementary Data 9

**Description:** *List of tandem stagger models from cross-link guided Rosetta molecular modelling satisfying cross-links across linker L12.* Given are model names, number of satisfied cross-links, satisfied crosslinks, Interface score (I_sc), angle between the docked dimeric fragments and rod shortening conveyed by each model.

**File Name:** Supplementary Data 10

**Description:** *List of tandem stagger models from cross-link guided Rosetta molecular modelling satisfying cross-links across linker L2.* Given are model names, number of satisfied cross-links, satisfied crosslinks, Interface score (I_sc), angle between the docked dimeric fragments and rod shortening conveyed by each model.

**File Name:** Supplementary Data 11

**Description:** *List of tandem stagger models from cross-link guided Rosetta molecular modelling satisfying cross-links across linker L3.* Given are model names, number of satisfied cross-links, satisfied crosslinks, Interface score (I_sc), angle between the docked dimeric fragments and rod shortening conveyed by each model.

**File Name:** Supplementary Data 12

**Description:** *List of tandem stagger models from unconstrained Rosetta molecular modelling satisfying cross-links across linker L1.* Given are model names, number of satisfied cross-links, satisfied crosslinks, Interface score (I_sc), angle between the docked dimeric fragments.

**File Name:** Supplementary Data 13

**Description:** *List of tandem stagger models from unconstrained Rosetta molecular modelling satisfying cross-links across linker L12.* Given are model names, number of satisfied cross-links, satisfied crosslinks, Interface score (I_sc), angle between the docked dimeric fragments.

**File Name:** Supplementary Data 14

**Description:** *List of tandem stagger models from unconstrained Rosetta molecular modelling satisfying cross-links across linker L2.* Given are model names, number of satisfied cross-links, satisfied crosslinks, Interface score (I_sc), angle between the docked dimeric fragments.

**File Name:** Supplementary Data 15

**Description:** *List of tandem stagger models from unconstrained Rosetta molecular modelling satisfying cross-links across linker L3.* Given are model names, number of satisfied cross-links, satisfied crosslinks, Interface score (I_sc), angle between the docked dimeric fragments.
