# Supplementary material for: Lamin A molecular compression and sliding as mechanisms behind nucleoskeleton elasticity
Source: Nat Commun. 2019 Jul 11;10:3056. doi: 10.1038/s41467-019-11063-6 (PMC6624373; doi:10.1038/s41467-019-11063-6)
Supplement: Supplementary file 19 — Reporting Summary [file 41467_2019_11063_MOESM19_ESM.pdf]

Corresponding author(s): Juri Rappsilber and Eric C. Schirmer

Last updated by author(s): Jun 13, 2019

# Reporting Summary

Nature Research wishes to improve the reproducibility of the work that we publish. This form provides structure for consistency and transparency in reporting. For further information on Nature Research policies, see Authors & Referees and the Editorial Policy Checklist.

## Statistics

For all statistical analyses, confirm that the following items are present in the figure legend, table legend, main text, or Methods section.

| n/a | Confirmed | |
|---|---|---|
| ☐ | ☒ | The exact sample size (*n*) for each experimental group/condition, given as a discrete number and unit of measurement |
| ☐ | ☒ | A statement on whether measurements were taken from distinct samples or whether the same sample was measured repeatedly |
| ☐ | ☒ | The statistical test(s) used AND whether they are one- or two-sided<br>*Only common tests should be described solely by name; describe more complex techniques in the Methods section.* |
| ☒ | ☐ | A description of all covariates tested |
| ☐ | ☒ | A description of any assumptions or corrections, such as tests of normality and adjustment for multiple comparisons |
| ☐ | ☒ | A full description of the statistical parameters including central tendency (e.g. means) or other basic estimates (e.g. regression coefficient) AND variation (e.g. standard deviation) or associated estimates of uncertainty (e.g. confidence intervals) |
| ☐ | ☒ | For null hypothesis testing, the test statistic (e.g. *F*, *t*, *r*) with confidence intervals, effect sizes, degrees of freedom and *P* value noted<br>*Give P values as exact values whenever suitable.* |
| ☒ | ☐ | For Bayesian analysis, information on the choice of priors and Markov chain Monte Carlo settings |
| ☒ | ☐ | For hierarchical and complex designs, identification of the appropriate level for tests and full reporting of outcomes |
| ☒ | ☐ | Estimates of effect sizes (e.g. Cohen's *d*, Pearson's *r*), indicating how they were calculated |

*Our web collection on statistics for biologists contains articles on many of the points above.*

## Software and code

Policy information about availability of computer code

| | |
|---|---|
| Data collection | No commercial/open source/custom code was used to collect raw wet experimental data in this study except the in-built software driving LTQ Orbitrap Velos and Orbitrap Fusion Lumos Tribrid Mass Spectrometers (Thermo Fisher Scientific), JEOL 1200 TEM (JEOL), ACE600 e-beam coater (Leica). Molecular modelling data was produced using ROSETTA downloadable build 2017.08.59291, MODELLER version 9.19 - both available under a Free Academic License - and SWISS-MODEL Workspace and I-Tasser public servers (https://swissmodel.expasy.org/ & https://zhanglab.ccmb.med.umich.edu/I-TASSER/). EPS .pqr files were calculated using PDB2PQR public servers (http://nbcr-222.ucsd.edu/pdb2pqr_2.1.1/) |
| Data analysis | Proteomics cross-linking data was analysed using publically available MaxQuant v1.5.3.30 and MSCovert v3.0.11417 (ProteoWizard) available under free licence. Cross-linked peptides were analysed using XiSearch engine v1.6.742 and XiFDR v1.1.27 publically available from Rappsilber group and Thermo Xcalibur software v4.0.27.1 (Thermo Fisher Scientific). Cross-linking data was visualised using xiNET engine (http://crosslinkviewer.org/index.php) publically hosted by Rappsilber group. Molecular modelling data was further analysed using ROSETTA downloadable build 2017.08.59291, freely available Xwalk software downloadable version 0.6 and Open-Source PyMOL 1.6.0.0. EM micrographs and protein gels were analysed in ImageJ. Western blot data was analysed in Image Studio version 2.1.10 (Li-Cor Biosciences). Statistical analysis was carried out in R public release v3.5.1. |

For manuscripts utilizing custom algorithms or software that are central to the research but not yet described in published literature, software must be made available to editors/reviewers. We strongly encourage code deposition in a community repository (e.g. GitHub). See the Nature Research guidelines for submitting code & software for further information.

## Data

Policy information about availability of data

All manuscripts must include a data availability statement. This statement should provide the following information, where applicable:

- Accession codes, unique identifiers, or web links for publicly available datasets
- A list of figures that have associated raw data
- A description of any restrictions on data availability

Data generated or analysed during the current study are included in the published manuscript and its supplementary information files. All the mass spectrometry proteomics data generated in this study (Figures 4-6, 8, 9) have been deposited to the ProteomeXchange Consortium via the PRIDE partner repository with the dataset identifier PXD008337 and PXD014009. Rosetta molecular modelling data is available via Edinburgh DataShare (https://datashare.is.ed.ac.uk/handle/10283/3348).

# Field-specific reporting

Please select the one below that is the best fit for your research. If you are not sure, read the appropriate sections before making your selection.

☒ Life sciences ☐ Behavioural & social sciences ☐ Ecological, evolutionary & environmental sciences

For a reference copy of the document with all sections, see nature.com/documents/nr-reporting-summary-flat.pdf

# Life sciences study design

All studies must disclose on these points even when the disclosure is negative.

| | |
|---|---|
| Sample size | Sample size for EM micrographs measurements were determined in line with previous publications and by the practical limitations of the protocols utilised. Sample size for other measurements were determined in line with convention of the method. No power analysis was preformed for the design of these studies. |
| Data exclusions | No outlier data points were excluded in this study. Full MS data was utilised after 5% FDR calculation. |
| Replication | Data described in this manuscript were reliably reproduced. Two MS experiments with homo- and homo-/hetero-iso-dimeric material served as controls for each other. |
| Randomization | No sample randomization was performed or required in this study. |
| Blinding | No blinding was used in this studies. |

# Reporting for specific materials, systems and methods

We require information from authors about some types of materials, experimental systems and methods used in many studies. Here, indicate whether each material, system or method listed is relevant to your study. If you are not sure if a list item applies to your research, read the appropriate section before selecting a response.

### Materials & experimental systems

| n/a | Involved in the study | |
|---|---|---|
| ☐ | ☒ | Antibodies |
| ☒ | ☐ | Eukaryotic cell lines |
| ☒ | ☐ | Palaeontology |
| ☐ | ☒ | Animals and other organisms |
| ☒ | ☐ | Human research participants |
| ☒ | ☐ | Clinical data |

### Methods

| n/a | Involved in the study | |
|---|---|---|
| ☒ | ☐ | ChIP-seq |
| ☒ | ☐ | Flow cytometry |
| ☒ | ☐ | MRI-based neuroimaging |

## Antibodies

| | |
|---|---|
| Antibodies used | Rabbit polyclonal antibody 5881 against a region [572-585] downstream of the Ig fold in the lamin A tail domain was used for Western Blot detection of cross-linked lamin A from rat liver. LICOR anti-rabbit IR800 antibody (Li-Cor Biosciences, #925-32213) was used as secondary. |
| Validation | 5881 antibody was validated in Yang, L., Guan, T. & Gerace, L. J Cell Biol 137, 1199-1210 (1997). |

## Animals and other organisms

Policy information about studies involving animals; ARRIVE guidelines recommended for reporting animal research

| | |
|---|---|
| Laboratory animals | Intact nuclear envelope material was obtained from livers of laboratory Sprague Dawley Rats (Rattus Norvegicus) |
| Wild animals | n/a |
| Field-collected samples | n/a |
| Ethics oversight | n/a |

Note that full information on the approval of the study protocol must also be provided in the manuscript.
